# Molecular basis for the biosynthesis of the siderophore coprogen in the cheese-ripening fungus *Penicillium roqueforti*

**DOI:** 10.1186/s40659-025-00633-2

**Published:** 2025-07-21

**Authors:** Kathia González, Mariana Montanares, Matías Gallardo, Carlos Gil-Durán, Abel M. Forero, Jaime Rodríguez, Carlos Jiménez, Inmaculada Vaca, Renato Chávez

**Affiliations:** 1 Departamento de Biología, Facultad de Química y Biología, Universidad de Santiago de Chile (USACH), Alameda 3363, Estación Central, Santiago Chile; 2 Departamento de Química, Facultad de Ciencias, Universidad de Chile, Las Palmeras 3425, Ñuñoa, Santiago Chile; 3 CICA-Centro Interdisciplinar de Química e Bioloxía, Departamento de Química, Facultade de Ciencias, Universidade da Coruña, 15071 A Coruña, Spain

**Keywords:** *Penicillium roqueforti*, Siderophore, Coprogen, Biosynthetic gene cluster, CRISPR-Cas9, Mass spectrometry

## Abstract

**Background:**

Iron is an essential nutrient for microorganisms, including fungi, which have evolved strategies to acquire it. The most common strategy is the secretion of siderophores, low-molecular-weight compounds with a high affinity for ferric ions, which are involved in cellular iron uptake. *Penicillium roqueforti*, the fungus responsible for the ripening of blue-veined cheeses, produces coprogen, a hydroxamate-type siderophore. However, to date, the molecular basis for its biosynthesis remains elusive.

**Results:**

In this study, we identified and characterized a biosynthetic gene cluster (BGC) responsible for coprogen biosynthesis in *P. roqueforti*, named the *cop* BGC. This BGC contains seven genes, three of which (*copA*, *copB* and *copE*) encode enzymes directly involved in coprogen biosynthesis from precursors molecules. Using CRISPR-Cas9, we targeted these three genes and analyzed the resulting mutants by Liquid Chromatography/High-Resolution Mass Spectrometry (LC/HRMS). Our results confirmed that all three genes are necessary for coprogen biosynthesis. Phenotypically, the mutants displayed growth differences under iron-deficient conditions, which correlated with their ability to either synthesize or fail to synthesize coprogen B and dimerumic acid, intermediates in the coprogen pathway with siderophore activity.

**Conclusions:**

The results obtained in this work provide important insights into the molecular basis of coprogen biosynthesis in *P. roqueforti*, enhancing the understanding of how siderophores enable this fungus to thrive in iron-deficient environments.

**Supplementary Information:**

The online version contains supplementary material available at 10.1186/s40659-025-00633-2.

## Background

Iron is an essential micronutrient for microorganisms, playing a crucial role in various physiological processes. This metal functions as a cofactor in enzymes involved in key cellular activities such as DNA replication, transcription, repair, and carbon metabolism [1]. Additionally, iron is a component of the prosthetic groups of metalloproteins, where it acts as a biocatalyst or electron carrier, with its redox properties being critical for electron transport in the respiratory chain [1].

Despite the essential biological roles of iron, this metal is not readily bioavailable in nature, even though it is one of the most abundant elements in Earth's crust [2]. Around 2.4 billion years ago, the massive accumulation of atmospheric oxygen produced by cyanobacteria (an event known as the Great Oxidation Event) led to the oxidation of iron from its ferrous (Fe^2^⁺) to ferric (Fe^3^⁺) form, which is now the predominant state of iron on Earth [3]. Ferric ions have poor solubility at physiological pH, significantly limiting their bioavailability [4, 5]. Consequently, microorganisms have evolved various strategies to acquire ferric ions from the environment [6, 7], with siderophore-mediated iron uptake being one of the most common [8, 9].

Siderophores are low-molecular-weight organic compounds produced by a wide range of microorganisms, including bacteria and fungi [10, 11]. These molecules have a high affinity for ferric ions, forming soluble complexes that are recognized by specific outer membrane protein transporters, which facilitate the internalization of iron into the cells [4, 11]. Based on their iron-coordinating groups, microbial siderophores are classified into three main categories: catecholates, carboxylates, and hydroxamates [4, 9]. In fungi, hydroxamate-type siderophores are the most frequently reported [9]. These hydrophilic molecules possess bidentate ligands, in which the oxygen atoms of the hydroxamate group coordinate tightly with ferric ions [4]. The main hydroxamate siderophores produced by fungi are fusarinines, coprogens, and ferrichromes [12].

The biosynthesis of hydroxamate siderophores involves different enzymatic pathways, most of which start with the common precursor *N*^5^-hydroxyornithine, a molecule formed by the hydroxylation of the amino group of ornithine [8, 10, 11]. *N*^*5*^-hydroxyornithine then undergoes acylation, with the specific acyl group determining the type of siderophore produced. For instance, the addition of acetyl-CoA leads to the formation of *N*^*5*^-acetyl-*N*^*5*^-hydroxyornithine, the precursor for ferrichrome biosynthesis, while the addition of anhydromevalonyl-CoA results in *N*^*5*^-anhydromevalonyl-*N*^*5*^-hydroxyornithine (AMHO), involved in the production of fusarinines and coprogens [8, 10, 11]. Subsequently, specific non-ribosomal peptide synthetase (NRPS) enzymes condense AMHO units to form fusarinine and coprogen siderophores, or *N*^*5*^-acetyl-*N*^*5*^-hydroxyornithine units along with serine and glycine to form ferrichrome-type siderophores. Further enzymatic modifications, such as acylation, methylation, or hydroxylation, contribute to the structural diversity of hydroxamate siderophores [8, 10, 11].

The filamentous fungus *Penicillium roqueforti* is one of the most emblematic species within the genus *Penicillium*. This fungus is distributed across a wide range of ecological niches, including both natural and anthropogenic environments, such as soil, spoiled foods, silage, lumber, and food products [13–15]. Among these habitats, one of the most well-known is cheese, particularly blue-veined cheese, where *P. roqueforti* plays a fundamental role in its ripening. Many strains of *P. roqueforti* have evolved physiological capabilities that make them well-adapted to the cheese environment [14, 15]. Thanks to these capacities, *P. roqueforti* thrives and becomes one of the dominant species in cheese [16].

An interesting characteristic of cheese is that it constitutes a highly iron-restricted environment. It has been estimated that iron concentrations in cheeses range between just 2 and 10 ppm [17]. This low iron content is due to the natural deficiency of iron in milk, which contains only 0.2–0.4 ppm [18], along with the presence of lactoferrin, an iron-binding protein that sequesters most of the iron that would otherwise be available to microorganisms in cheese [17, 18]. Consequently, microorganisms inhabiting cheeses, like *P. roqueforti*, have evolved mechanisms to compete for iron acquisition.

The current knowledge of the mechanisms underlying siderophore biosynthesis in *P. roqueforti* is still quite limited. Previous investigations have demonstrated that *P. roqueforti*, cultivated on synthetic media under controlled laboratory conditions, produces hydroxamate-type siderophores [19]. Among the siderophores identified in *P. roqueforti*, coprogen is the most frequently reported. This hydroxamate-type siderophore was purified from culture broths of *P. roqueforti* and structurally characterized through NMR spectroscopy [17]. Furthermore, several studies have reported the presence of coprogen in cheeses ripened with *P. roqueforti* [17, 20]. These studies unequivocally demonstrate that *P. roqueforti* produces coprogen. However, the molecular mechanisms underlying its biosynthesis in this fungus remain unknown. Elucidating these mechanisms would be particularly valuable for understanding how coprogen enables *P. roqueforti* to thrive in its ecological niches, especially in iron-deficient environments. Consequently, to gain insights into the molecular basis of coprogen biosynthesis in this organism, we herein report the identification and functional analysis of the biosynthetic gene cluster (BGC) responsible for coprogen production in *P. roqueforti*.

## Methods

### Fungal strain and growth conditions for routine maintenance

In this study, the *P. roqueforti* strain CECT 2905 was utilized. This strain is derived from the neotype of the species, which was originally isolated from Roquefort cheese [21]. For routine maintenance, the fungus was cultured on potato dextrose agar (PDA) at 28 °C. Additional culture conditions and media for specific experiments are detailed in the following sections.

### Identification of the BGC responsible for coprogen biosynthesis in *P. roqueforti*

The FASTA file containing the genome sequence of *P. roqueforti* CECT 2905 [21] was utilized to generate the genomic annotation file (GFF format) employing the Augustus software [22] with default parameters. For this analysis, Augustus was previously trained using the annotated genome of *P. roqueforti* strain FM164 [23]. Subsequently, both the genome and annotation files were used for the identification of the BGC associated with coprogen biosynthesis through antiSMASH fungal version 7.1.0 [24]. The analysis with antiSMASH was conducted with the “detection strictness” parameter set to “strict”, and the “extra features” parameter enabled for “All on”. Following the detection, the BGC was manually delimited through BlastP analysis. Finally, the closest homologue proteins from characterized BGCs were identified using the “MiBIG Hits” and “Blast against antiSMASH-database” tools, both integrated within antiSMASH fungal version 7.1.0. The BGC responsible for coprogen biosynthesis in *P. roqueforti* described in this work has been deposited in the GenBank database under accession number PQ588424.

### Disruption of genes of interest in the coprogen BGC using CRISPR-Cas9

The genes hypothesized to encode proteins involved in the coprogen biosynthetic pathway were disrupted in *P. roqueforti* using CRISPR-Cas9, as recently described by Marcano et al. [25]. Briefly, suitable target DNA sequences of each gene were identified using the CHOPCHOP program [26], which includes the genome of *P. roqueforti* in its database. Subsequently, suitable cassettes for the transcription of a sgRNA containing the specific target sequence were synthesized by Integrated DNA Technologies (IDT, USA). The structure of these cassettes, described in detail in a previous article [25], allows the transcription of a sgRNA specific to *P. roqueforti*.

The cassettes were subsequently cloned into the plasmid pFC333 [27] using suitable restriction sites. The pFC333 plasmid is equipped with all necessary elements for the expression of a Cas9 enzyme optimized for translation in fungal systems. Additionally, pFC333 contains an autonomous origin of replication (the AMA1 region), allowing for autonomous replication and avoiding genomic integration, as well as a gene conferring resistance to phleomycin, which enables the selection of fungal transformants [27]. The resulting plasmids were introduced into *P. roqueforti* following a standard protoplast-mediated transformation protocol established in our laboratory [28]. The transformants obtained were grown on different media to obtain monokaryotic strains and induce the loss of phleomycin resistance, as previously described by Marcano et al. [25]. Finally, successful disruption of each target gene was confirmed through direct sequencing of the respective target regions.

### Siderophore production and high-resolution mass spectrometry analysis

Previous research demonstrated that siderophores from *P. roqueforti* are well-produced when the fungus is cultured in synthetic modified M9 (MM9) medium [19]. In the present study, we utilized the same medium with slight modifications. A suspension of 1 × 10^8^ spores of the fungal strains of interest, grown for 5 days at 28 °C on PDA plates, was inoculated into flasks containing 50 mL of MM9. The composition of MM9 was as follows: Na_2_HPO_4_·2H_2_O (12.8 g/L), sucrose (10 g/L), KH_2_PO_4_ (3 g/L), NH_4_Cl (1 g/L), MgSO_4_·7H_2_O (0.65 g/L), NaCl (0.5 g/L), MgCl_2_·6H_2_O (61.3 mg/L), CaCl_2_ (11.1 mg/L), CaCO_2_ (2.5 mg/L), ZnSO_4_·7H_2_O (1.8 mg/L), MnSO_4_·H_2_O (1.1 mg/L), CuSO_4_·5H_2_O (0.3 mg/L), thiamine (0.3 mg/L), and H_3_BO_3_ (0.08 mg/L). To ensure effective iron depletion, the medium was supplemented with 100 µM bathophenanthroline disulfonic acid (BPS), a strong iron chelator. Cultures were incubated for 7 days and 28 °C, under agitation at 180 rpm.

Following incubation, cultures were filtered using Miracloth, and the resulting filtrate was treated by the addition of 5 mg FeCl_3_. The mixture was incubated overnight at 4 °C and subsequently lyophilized. The lyophilized material was resuspended in 40 mL of methanol, sonicated for 15 min, and centrifuged at 9000 rpm for 10 min. The supernatant (methanolic phase) was evaporated to dryness using a rotary evaporator and subsequently resuspended in 1 mL of LC/MS grade methanol. Finally, the supernatant was filtered through a 0.22 µm filter, and 5 µL of the sample were injected into a liquid chromatography system coupled to a high-resolution mass spectrometer (LC/HRMS).

LC/HRMS analysis was conducted on a UPLC-QTOF-ESI–MS system (Waters Xevo G2-XS QTof/Tof, USA) equipped with an electrospray ionization (ESI) source, a binary pump, an autosampler, and Waters MassLynx™ software. Chromatographic separation was performed using an ACQUITY UPLC BEH C18 column (1.7 µm particle size). The mobile phases consisted of phase A (0.1% formic acid in water) and phase B (0.1% formic acid in acetonitrile), with the following gradient elution profile: 5% solvent B from 0 to 1 min, followed by linear increase from 5 to 100% solvent B over 10 min, maintained isocratically for 2 min, and then returning linearly from 100 to 5% solvent B over 2 min. The flow rate was set to 0.3 mL/min, with the column maintained at a temperature of 40 °C.

Mass spectrometry data were acquired in positive ion mode over a mass range of *m/z* 50–1200. The MS parameters were as follows: nitrogen was used as the desolvation gas at a flow rate of 600 L/h, with a desolvation temperature of 350 °C. The capillary voltage was set to 3.5 kV, and the cone voltage was 40 V.

### Growth of *P. roqueforti* strains under iron-limited conditions and Chrome Azurol S (CAS) agar plate assay for siderophore detection

The growth of *P. roqueforti* strains under iron-limited conditions was assessed on agar plates containing MM9 medium supplemented with 100 µM BPS (prepared as described in the previous section) and 1.5% agar. Each plate was inoculated at center with 1 × 10^6^ spores of the fungal strain of interest and incubated at 28 °C for 7 days. At the end of the incubation period, colony diameters were measured, averaged, and plotted to assess growth under iron-limited conditions.

For siderophore detection, CAS agar plates were prepared according to the protocol of Louden et al. [29], with minor modifications. Specifically, 32.24 g piperazine-*N,N′*-bis(2-ethanesulfonic acid) (PIPES) was dissolved in 900 mL of MM9 medium (see the previous section for composition) supplemented with 100 µM BPS and adjusted to pH 6.2. The medium was then mixed with 15 g of agar and autoclaved. Separately, the blue dye solution, consisting of the ternary complex CAS/iron(III)/hexadecyltrimethylammonium bromide (CAS/Fe^3^⁺/HDTMA), was prepared as described by Louden et al. [29] and autoclaved. Once both solutions were cooled to 50 °C, they were combined in a 9:1 ratio (MM9 agar:blue dye solution) and poured into sterile Petri dishes. For the assay, 1 × 10^6^ spores of the fungal strain were placed at the center of each plate and incubated for 7 days at 28 °C. Siderophore production was evidenced by the formation of an orange halo around the fungal colony, representing iron chelation. The extent of siderophore production was measured by calculating the siderophore index, which is the ratio of the orange halo diameter to the fungal colony diameter, as previously described [30, 31].

## Results

### Identification and bioinformatic analysis of a BGC responsible for coprogen biosynthesis in *P. roqueforti*

**Fig. 1:**
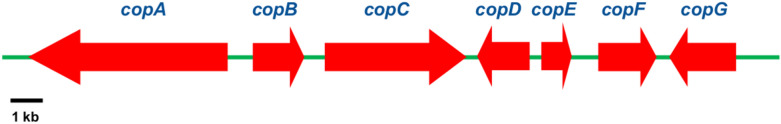
Schematic representation of the *cop* BGC identified in *P. roqueforti*. The genes are represented by *red arrows* in the 5′ to 3′ orientations. Details of the predicted proteins encoded by these genes are provided in Table 1

The genomic analysis of *P. roqueforti* CECT 2905 using antiSMASH fungal version identified a region with 37% overall nucleotide similarity to the BGC responsible for producing dimerumic acid and metachelins (mannosylated and *N*-oxidized coprogen-type siderophores) in the fungus *Metarhizium robertsii* [32]. This region, located within contig MSQC01000321 of the *P. roqueforti* CECT 2905 genome, was further manually analyzed and delimited using BlastP. The resulting gene cluster, named as the *cop* BGC, has a length of 20,450 bp and encompasses seven genes, from *copA* to *copG* (Fig. 1). Table 1 summarizes the main characteristics of the proteins encoded by these genes.

**Table 1:** Analysis of the deduced proteins encoded by the *cop* BGC of *P. roqueforti*

Name of the gene	Size of the deduced protein (aminoacids)	Putative function in coprogen biosynthesis	Closest homologue protein from a characterized BGC, according to “MiBIG Hits” tool	GenBank accession number of homologue protein	Identity (%) with homologue protein
*copA*	1898	Non-ribosomal peptide synthase (NRPS)	mrSidD from *Metarhizium robertsii*	EFY99276.1	52
*copB*	442	Acyl-CoA *N*-acyltransferase	Siderophore biosynthesis protein from *Metarhizium robertsii*	EFY99278.1	49
*copC*	1322	Transporter	AtrH like protein from *Metarhizium robertsii*	EFY99280.1	53
*copD*	416	Unknown	Pyridine nucleotide-disulfide oxidoreductase, NAD-binding domain protein from *Metarhizium robertsii*	EFY99279.1	45
*copE*	233	Acyl-CoA *N*-acyltransferase	acyl-CoA *N*-acyltransferase from *Shiraia* sp.	AHG26155	35
*copF*	505	Unknown	Aryl sulfotransferase LipB from *Streptomyces* sp.	BAJ05878.1	33
*copG*	586	Transporter	MFS transporter from *Byssochlamys fulva*	ANF07276.1	39

Analysis using the “MiBIG Hits” and “Blast against antiSMASH-database” tools identified four genes within this BGC (*copA*, *copB*, *copC* and *copD*) that encode proteins with high sequence similarity to those deduced from the corresponding BGC in *M. robertsii* [32] (Table 1). On the other hand, CopE, CopF and CopG exhibit moderate identity (33 to 39%, Table 1) to proteins encoded by BGCs involved in the biosynthesis of huperzine A in *Shiraia* sp. [33], the fatty acid nucleoside antibiotic A-90289 in *Streptomyces* sp. [34], and the mycotoxin byssochlamic acid in *Byssochlamys fulva* [35], respectively.

**Fig. 2:**
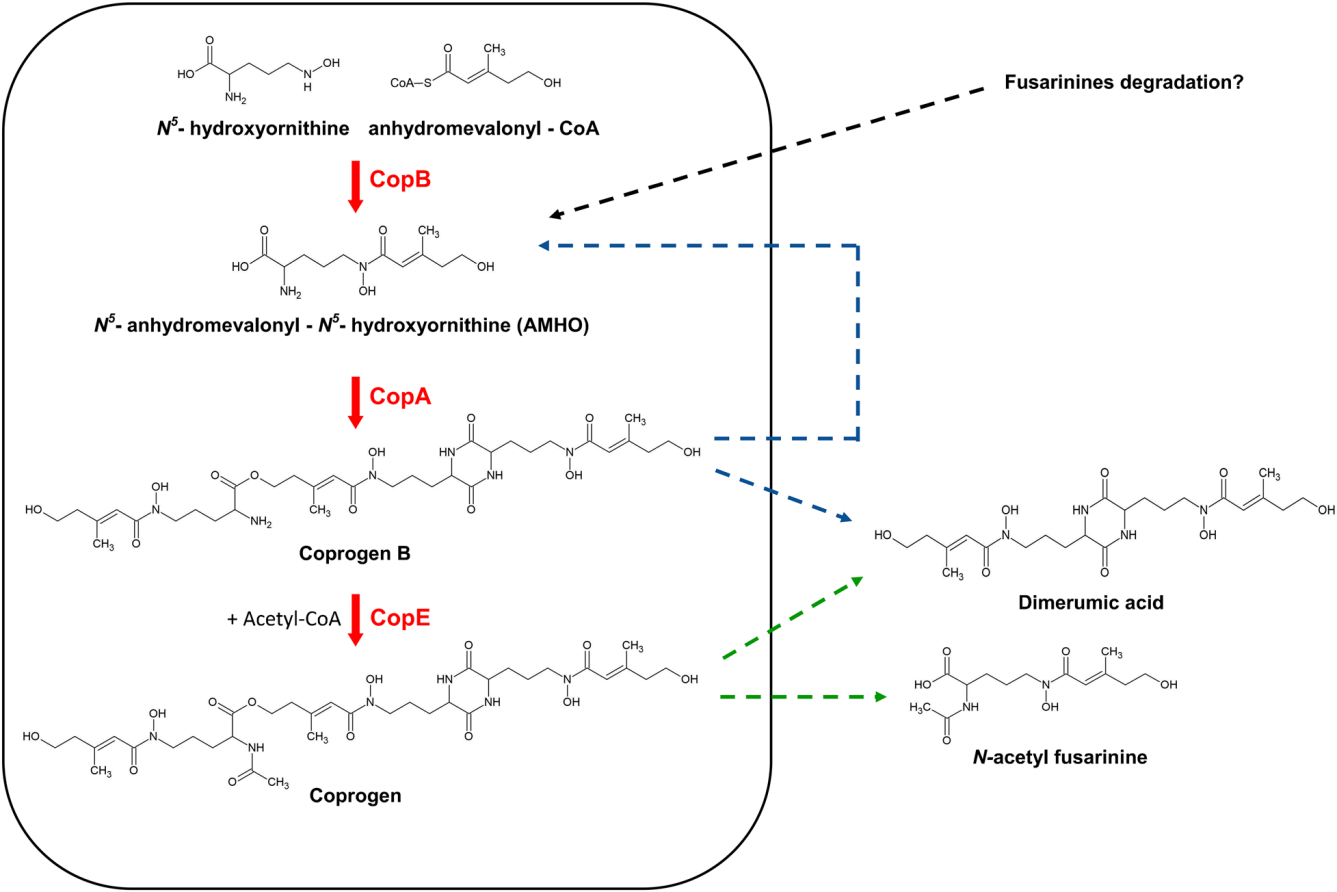
Proposed biosynthetic pathway for coprogen production in *P. roqueforti*. The core biosynthetic route is highlighted within the *black box*. *Dashed arrows* indicate proposed degradation pathways. The *black dashed arrow* represents the putative origin of AMHO in the copB-12 mutant, potentially resulting from fusarinines degradation. *Blue dashed arrows* denote the proposed conversion of the intermediate coprogen B into AMHO and dimerumic acid. *Green dashed arrows* indicate the degradation of coprogen into dimerumic acid and *N*-acetyl fusarinine

According to the literature, the biosynthesis of coprogen from the precursors *N*^*5*^-hydroxyornithine and anhydromevalonyl-CoA requires three consecutive enzymatic steps, catalyzed by an acyl-CoA *N*-acyltransferase, a specialized NRPS, and a second acyl-CoA *N*-acyltransferase [8, 10, 11, 36]. Based on the predicted enzymatic functions detailed in Table 1, the genes *copA*, *copB* and *copE* are proposed to encode these key enzymes involved in the coprogen biosynthesis in *P. roqueforti*. A hypothetical biosynthetic pathway illustrating the roles of these enzymes is presented in Fig. 2. Accordingly, the functional roles of *copA*, *copB* and *copE* were investigated in subsequent experiments.

### CRISPR-Cas9-mediated disruption of *copA*, *copB*, and *copE* and its impact on coprogen production in *P. roqueforti*

**Fig. 3:**
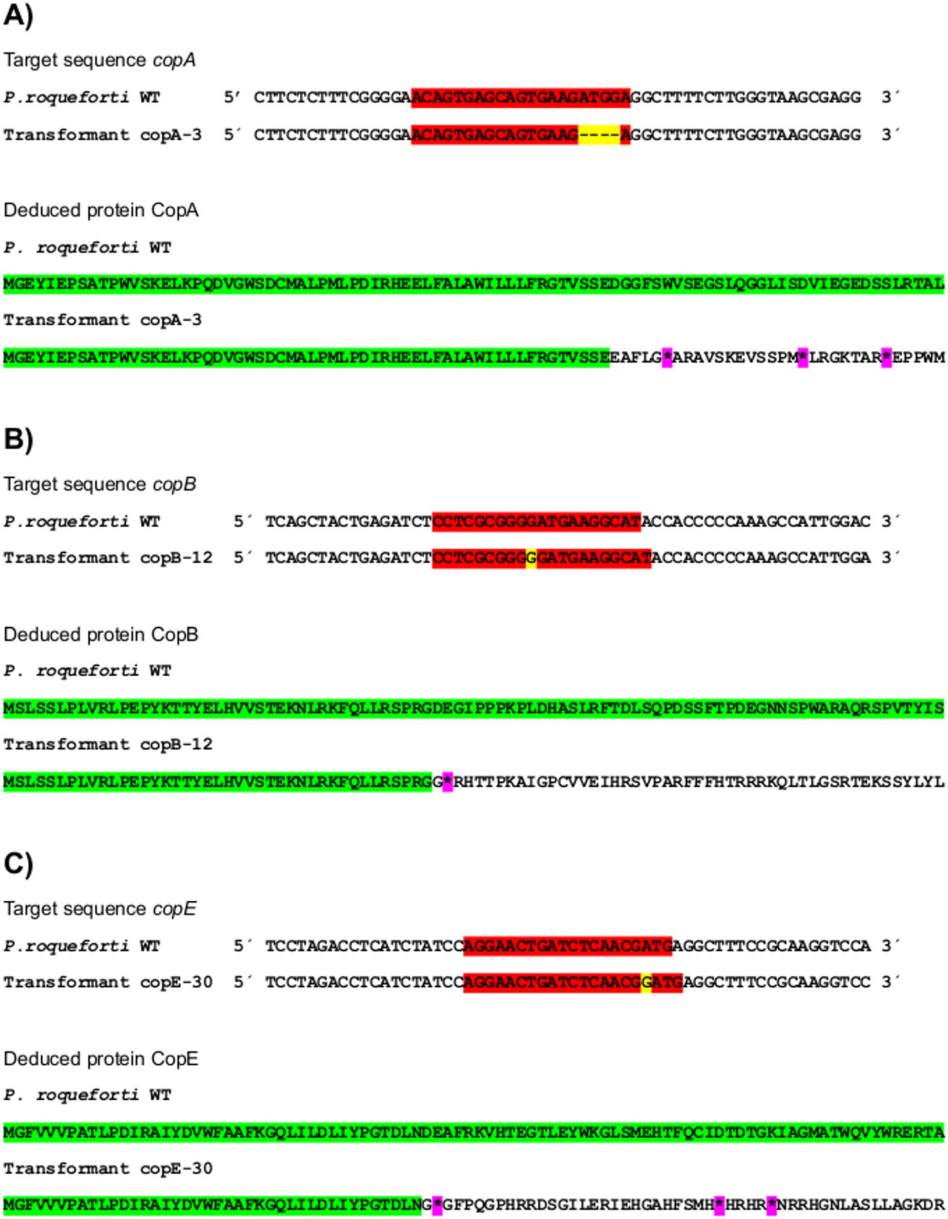
Comparison of the nucleotide sequences of the CRISPR-Cas9 target regions and the corresponding deduced amino acid sequences for *P. roqueforti* wild-type (WT) and the disrupted transformants copA-3 (**A**), copB-12 (**B**) and copE-30 (**C**). CRISPR-Cas9 target sites are marked in *red*, and induced insertions or deletions are highlighted in *yellow*. The amino end of each protein is shown, with regions unaffected by CRISPR-Cas9 highlighted in *green*. Subsequent regions exhibiting frameshifts are unmarked, and examples of premature stop codons are indicated by *asterisks in pink*

To functionally characterize the roles of *copA*, *copB*, and *copE* genes in coprogen biosynthesis, these genes were disrupted using the CRISPR-Cas9 system. Following the transformation of *P. roqueforti* with suitable plasmids, multiple transformants were obtained for each gene of interest. The successful disruption of the target genes was verified by sequencing in several transformants. Three representative transformants (namely copA-3, copB-12 and copE-30) were selected for further experiments. These transformants display insertions or deletions of bases within the target sequences, leading to protein frameshifts and the generation of premature stop codons, thus effectively inactivating the respective genes (Fig. 3).

**Fig. 4:**
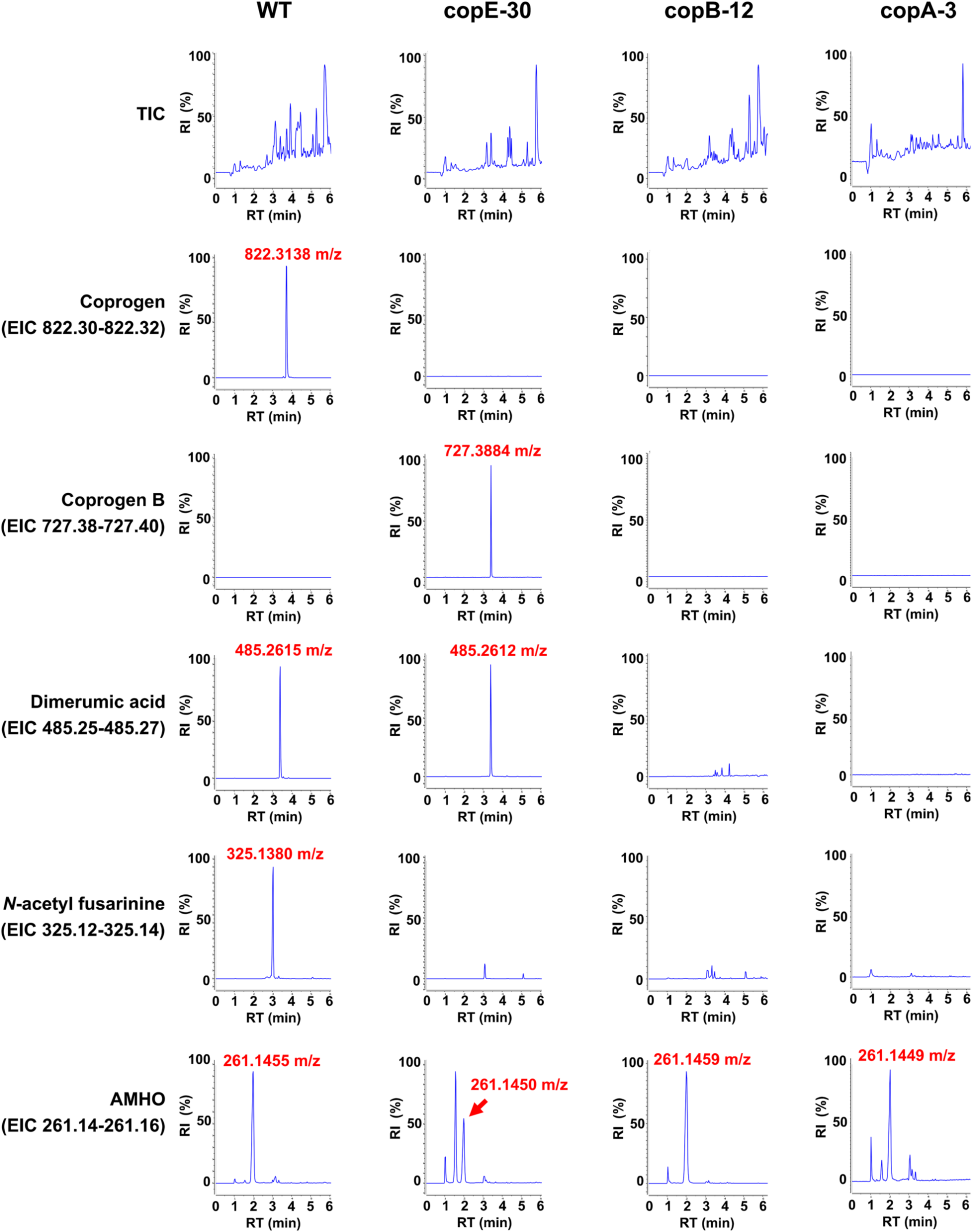
Identification of compounds from the coprogen biosynthetic pathway using UHPLC/MS in *P. roqueforti* wild-type (WT) and disrupted transformants copE-30, copB-12, and copA-3. The *top panels* display the total ion current (TIC) of extracts of each strain, while the subsequent panels show the extracted ion chromatograms (EICs) for specific ions corresponding to each compound of interest. The *m/z* values for each identified ion are labeled in *red* above the respective peaks. In all cases, the mass error between the measured and theoretical mass was less than 5 ppm. Further details are provided in the main text

Subsequently, the metabolic profiles of the mutants were analyzed using LC/HRMS and compared to that of the wild-type *P. roqueforti* strain under identical experimental conditions. Consistent with previous studies [17, 19], coprogen was clearly detected in the wild-type strain. Specifically, as depicted in Fig. 4, the ferric form of coprogen (*m/z* 822.3138, calc. for C_35_H_54_FeN_6_O_13_^+^ 822.3087 [M+Fe-2H]^+^) was identified in extracts from the wild-type strain at a retention time of 3.72 min. Additionally, the expected degradation products of coprogen were also present in this sample, including dimerumic acid (*m/z* 485.2615, [M+H]^+^, calc. for C_22_H_37_N_4_O_8_^+^ 485.2606) at a retention time of 3.40 min and *N*-acetyl fusarinine (*m/z* 325.1380 calc. for C_13_H_22_N_2_NaO_6_^+^ 325.1370 [M+Na]^+^) at a retention time of 3.04 min (Fig. 4). In contrast, the copA-3, copB-12, and copE-30 transformants did not show coprogen production (Fig. 4), thereby confirming that *copA*, *copB* and *copE* are essential genes in the biosynthetic pathway of this siderophore.

Based on bioinformatic analysis (Table 1), *copE* encodes an acyl-CoA *N*-acyltransferase predicted to catalyze the acetylation of coprogen B to form coprogen, which represents the final step in the biosynthetic pathway [10, 36]. LC/HRMS analysis of extracts from the copE-30 transformant confirmed this prediction. Inactivation of *copE* led to the absence of coprogen and the accumulation of a compound with *m/z* 727.3884 [M+H]^+^ (calc. for C_33_H_55_N_6_O_12_^+^ 727.3872) at a retention time of 3.42 min (Fig. 4). This compound, corresponding to the precursor coprogen B, was absent in extracts from wild-type *P. roqueforti* and the copA-3 and copB-12 transformants (Fig. 4). Additionally, broths from the copE-30 transformant contained dimerumic acid (*m/z* 485.2615, [M+H]^+^) at a retention time of 3.40 min but lacked *N*-acetyl fusarinine (Fig. 4), consistent with the expected degradation pattern for coprogen B [37].

For the case of *copB*, bioinformatic analysis predicts that this gene encodes the acyl-CoA *N*-acyltransferase that functions upstream in the biosynthetic pathway, catalyzing the condensation of ornithine and anhydromevanolyl-CoA to produce AMHO (Table 1). This prediction is supported by the observation that disruption of *copB* in the copB-12 transformant led to the complete absence of coprogen, *N*-acetyl fusarinine, coprogen B and dimerumic acid in the culture broths (Fig. 4). However, despite the lack of these expected downstream products, AMHO (*m/z* 261.1459, calc. for C_11_H_21_N_2_O_5_^+^ 261.1445 [M+H]^+^) was still detected in this mutant at a retention time of 2 min. This unexpected finding suggests that AMHO may be synthesized via an alternative biosynthetic route, independent of *copB*. As will be discussed in more detail later, this could involve a distinct acyl-CoA *N*-acyltransferase encoded by a separate fusarinine-type BGC identified in the *P. roqueforti* genome (Additional file 1). The presence of this alternate pathway may account for the AMHO accumulation observed in the copB-12 mutant, despite disruption of the canonical coprogen biosynthetic route.

Finally, the copA-3 mutant was analyzed. According to bioinformatic analysis (Table 1), *copA* encodes the NRPS within the pathway. Thus, its disruption would be expected to result in the absence of coprogen, *N*-acetyl fusarinine, coprogen B and dimerumic acid. As shown in Fig. 4, none of these metabolites were detected in extracts from the copA-3 transformant, where *copA* was disrupted.

Taken together, the previous findings suggest that *copB* encodes the acyl-CoA *N*-acyltransferase responsible for the acylation of *N*^*5*^-hydroxyornithine with anhydromevalonyl-CoA to form AMHO, *copA* encodes the NRPS involved in the biosynthesis of coprogen B from AMHO precursor units, and *copE* encodes the second acyl-CoA *N*-acyltransferase that catalyzes the acetylation of coprogen B to yield coprogen. These findings are consistent with the biosynthetic pathway proposed in Fig. 2, which also depicts likely routes for the formation of the degradation products detected in the LC/HRMS analyses.

### Differential growth of disrupted mutants under iron-limited conditions supports the proposed biosynthetic pathway

**Fig. 5:**
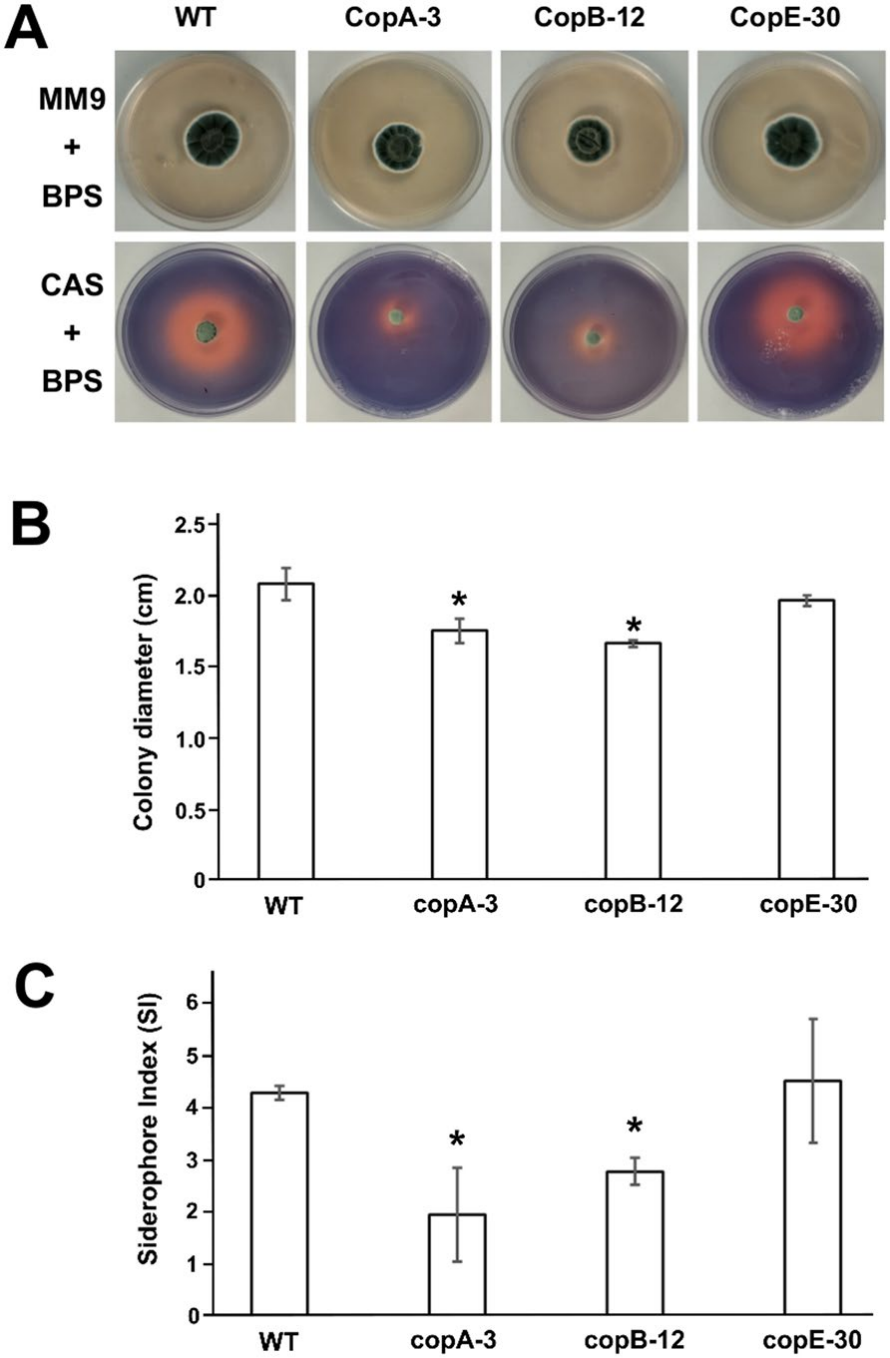
**A** Growth of *P. roqueforti* wild-type (WT) and disrupted and mutants copA-3 copB-12 and copE-30 on MM9 plates supplemented with 100 µM BPS after 7 days. The same strains are also shown on CAS plates, where the *orange* halos around the colonies indicate siderophore activity. **B** Quantification of colony diameter (cm) for each strain grown on MM9 plates with 100 µM BPS after 7 days. *Asterisks* denote statistically significant differences in growth compared to WT strain (*p* < 0.05, Student’s *t*-test). **C** Siderophore index (SI) for *P. roqueforti* wild-type (WT) and the disrupted mutants copA-3 copB-12 and copE-30. *Asterisks* indicate statistically significant differences in SI compared to WT strain (*p* < 0.05, Student’s *t*-test)

LC/HRMS analysis of the disrupted mutants was highly valuable in elucidating the biosynthetic pathway for coprogen in *P. roqueforti*. Furthermore, the experiments enabled the verification of the absence or presence of various intermediate and degradation products of the pathway within the mutants (Fig. 4). Notably, among these intermediate and degradation products, the siderophore activity of coprogen B and dimerumic acid has been previously reported [38–40]. Consequently, it was of interest to investigate whether the presence or absence of key intermediates with siderophore activity in the mutants correlates with differences in the growth of *P. roqueforti* under iron-limited conditions. To address this, we analyzed the growth of *P. roqueforti* CECT 2905 (wild-type strain) and the transformants copA-3, copB-12, and copE-30 in MM9 medium supplemented with 100 µM BPS to deplete iron (Fig. 5A).

In general, all mutants deficient in coprogen production exhibited growth in iron-limited media (Fig. 5A). However important differences were observed among the mutants. Specifically, mutants copA-3 and copB-12 showed significantly impaired growth. The colony diameter of wild-type *P. roqueforti* after 7 days ranged from 1.96 to 2.16 cm, whereas the average colony diameters for mutants copA-3 and copB-12 were 1.76 and 1.67 cm, respectively, indicating a significant reduction in growth compared to the wild-type strain under identical conditions (Fig. 5A, B). In contrast, although mutant copE-30 showed a slight decrease in growth, this reduction was not statistically significant when compared to the wild-type strain (Fig. 5A, B).

The previous findings correlated with the observed siderophore production in both the wild-type *P. roqueforti* and the mutants copA-3, copB-12, and copE-30. As shown in the CAS plate assay (Fig. 5A), siderophore production was markedly reduced in the transformants copA-3 and copB-12 compared to the wild-type strain. The wild-type *P. roqueforti* CECT 2905 exhibited a high siderophore index (average 4.31), while mutants copA-3 and copB-12 showed significant reductions in their siderophore indices (averaging 1.95 and 2.70, respectively) (Fig. 5C). Conversely, mutant copE-30 did not show a substantial reduction in siderophore production in the plates assay relative to *P. roqueforti* CECT 2905 (Fig. 5A), with an average siderophore index (average 4.54) closely resembling that of the wild-type fungus (Fig. 5C).

## Discussion

The filamentous fungus *Penicillium roqueforti* plays a key role in the food industry, particularly in the ripening of blue cheeses. Despite its relevance, various biological aspects of this organism remain unexplored. While it has been known for many years that *P. roqueforti* produces the siderophore coprogen for iron uptake [17], the molecular basis of its biosynthesis has, until now, remained elusive. In the present study, we used a combination of bioinformatics, molecular biology, and high-resolution mass spectrometry techniques to identify and characterize the coprogen biosynthetic gene cluster (the *cop* BGC), responsible for synthesizing coprogen in *P. roqueforti*. The findings of this study provide valuable molecular insights into the biosynthesis of this siderophore.

According to the literature, the biosynthesis of coprogen requires the initial acylation of *N*^*5*^-hydroxyornithine by anhydromevalonyl-CoA to form AMHO, a reaction catalyzed by an acyl-CoA *N*-acyltransferase [8, 10, 11]. The resulting AMHO units are then condensed by a specialized NRPS to produce the intermediate coprogen B [10, 11]. In the final step of the pathway, coprogen B is acetylated by a second acyl-CoA *N*-acyltransferase to yield coprogen [10, 36]. Our results show that three genes within the *cop* BGC in *P. roqueforti* (*copA*, *copB*, and *copE*) encode the NRPS and two acyl-CoA *N*-acyltransferases required for coprogen biosynthesis. Based on these results, we propose a biosynthetic pathway for coprogen in *P. roqueforti* (Fig. 2). This pathway is consistent with the proposed mechanisms for coprogen and coprogen-type siderophore biosynthesis in other fungi [11, 32, 41–43].

In addition to the three biosynthetic genes that have been functionally characterized, the *cop* BGC includes four additional genes (*copC*, *copD*, *copF*, and *copG*) whose functions remain uncharacterized. Among them, *copG* encodes a predicted transporter belonging to the major facilitator superfamily (MFS). Members of this superfamily, particularly those classified within the siderophore iron transporter (SIT) subfamily, have been extensively implicated in the uptake of hydroxamate-type siderophore-iron complexes in fungi [10, 11]. Based on this functional association, it is plausible to hypothesize that CopG participates in the uptake of iron-bound coprogen complexes.

In contrast, *copC* encodes an ABC transporter. Although ABC transporters have been associated with iron homeostasis in fungi [44–46], their direct involvement in siderophore transport remains poorly supported. To date, the only fungal ABC transporter functionally linked to siderophore transport is AbcB in *Aspergillus fumigatus*, which does not directly mediate siderophore transport but rather facilitates the excretion of siderophore degradation products [47]. Based on this limited evidence, we speculate that CopC may participate in the export of coprogen degradation products to the extracellular environment.

Regarding *copF* and *copD*, the absence of experimental evidence supporting their involvement in siderophore biosynthesis makes it difficult to assign specific functions. Their annotations (Table 1) suggest potential involvement in sulfur-related modifications within the coprogen biosynthetic pathway. On one hand, *copF* encodes a putative sulfotransferase, an enzyme that catalyzes the transfer of a sulfate group from a donor molecule to alcohol or amine acceptors [48]. While relatively few sulfotransferases have been characterized in fungi, those that have been studied are often involved in the sulfation of intermediates in secondary metabolism, potentially altering solubility, stability, or biological activity [48, 49]. On the other hand, *copD* encodes a pyridine nucleotide-disulfide oxidoreductase. These enzymes catalyze redox reactions involving the interconversion between disulfide bonds and thiol groups, utilizing pyridine nucleotides as cofactors [50]. Interestingly, disulfide oxidoreductases have been associated with the formation or rearrangement of disulfide bonds in certain fungal secondary metabolites such as gliotoxin, sirodesmin, and the depsipeptide FK228 [51, 52]. The presence of these two sulfur-related enzymes within the *cop* BGC suggests that the coprogen biosynthetic pathway may involve sulfation or disulfide bond formation and rearrangement at certain stages. These modifications could be necessary for activating specific intermediates, enhancing structural diversity, or modulating the biological activity of the coprogen scaffold. Further studies are necessary to test these hypotheses and clarify the roles of *copD* and *copF* in the pathway.

In fungi, siderophore biosynthesis is tightly regulated by iron availability through conserved transcriptional regulators, particularly the GATA-like repressor SreA and the bZIP-type transcription factor HapX [7, 53, 54]. These factors maintain iron homeostasis by balancing iron uptake and storage, thereby preventing both deficiency and toxicity of this metal [7, 53, 54]. In *A. fumigatus*, SreA represses siderophore biosynthetic genes under iron-rich conditions, whereas HapX induces their expression under iron-limiting conditions [54, 55]. Notably, HapX can antagonize the repressive function of SreA, establishing a finely tuned regulatory balance in iron homeostasis [7, 54].

In *P. roqueforti*, these regulatory mechanisms have not yet been characterized. However, based on the models developed for *Aspergillus*, it is reasonable to speculate that a similar regulatory scheme might be present. Orthologs of *A. fumigatus* SreA and HapX have been identified in *P. roqueforti* (e.g., GenBank accession numbers CDM30849 and CDM35844), suggesting their potential involvement in the transcriptional regulation of the *cop* BGC. According to this hypothetical model, the HapX ortholog would activate the *cop* BGC under iron-deficient conditions, possibly by binding to conserved motifs in the promoter regions of target genes, while the SreA ortholog would repress BGC expression under iron-rich conditions to prevent unnecessary siderophore production. Although this regulatory model remains speculative, it provides a valuable framework for future studies aimed at elucidating the transcriptional control of the *cop* BGC in *P. roqueforti*.

An interesting result of our research was that all mutants deficient in coprogen production were able to grow in iron-limited media (Fig. 5A), indicating that *P. roqueforti* possesses alternative mechanisms for iron assimilation that support fungal viability. The mechanisms of iron assimilation in fungi are diverse and well-documented in literature. Beyond siderophore-mediated uptake, these strategies include reductive iron assimilation, direct transport of iron ions through specific transporters, and uptake of heme and hemoglobin, among others [6, 56–58]. However, despite the general growth capacity under iron-limited conditions, important differences were observed among the mutants. Thus, mutants copA-3 and copB-12 showed significantly impaired growth, while mutant copE-30 exhibited growth more in line with the wild-type strain (Fig. 5B). Notably, these differences in growth correlated with differences in the siderophore production, with mutants copA-3 and copB-12 showing significant reductions in their siderophore capacity, while mutant copE-30 did not show a substantial reduction in siderophore production (Fig. 5C). Collectively, these results corroborate the proposed biosynthetic pathway for coprogen depicted in Fig. 2. Transformants copA-3 and copB-12, which harbor disruptions in the *copA* and *copB* genes, respectively, are unable to produce coprogen and its intermediates with siderophore capacity, namely coprogen B and dimerumic acid (Fig. 4), resulting in their impaired growth, as observed in Fig. 5A. In contrast, although the disruption of the *copE* gene in transformant copE-30 precludes the production of coprogen, this strain retains the ability to synthesize coprogen B and the degradation product dimerumic acid (Fig. 4), both of which possess siderophore activity and may compensate for the coprogen deficiency, thereby supporting a growth more comparable to that of the wild-type *P. roqueforti*.

During the analysis of culture broths, we observed the consistent presence of AMHO in all mutants and the wild-type *P. roqueforti* strain (Fig. 4). Previous studies have shown that the accumulation of AMHO in culture broths is common in fungi such as *P. chrysogenum*, *Trichoderma* spp., and *Gliocladium virens* [37, 38, 59], although the biological significance of this accumulation remains unclear. Our findings suggest that *P. roqueforti* follows a similar pattern. As deduced from Fig. 2, AMHO present in the broths of both wild-type *P. roqueforti* and the copA-3 and copE-30 mutants may derive from the coprogen pathway, either as a degradation product of coprogen B or as a biosynthetic intermediate. However, this does not account for the presence of AMHO in the copB-12 transformant, where the coprogen pathway was interrupted upstream of AMHO production (Fig. 2). Therefore, in this transformant, AMHO must originate from an alternative source.

AMHO, in its *cis* configuration, can also be synthesized via the fusarinine biosynthetic pathway [8, 11], where a distinct acyl-CoA *N*-acyltransferase, different from the one involved in the coprogen pathway, is likely responsible for AMHO formation. To identify this alternative acyl-CoA *N*-acyltransferase, we analyzed the *P. roqueforti* genome using antiSMASH and identified a six-gene BGC potentially responsible for producing a fusarinine-type siderophore in *P. roqueforti* (see Additional file 1), likely fusarinine C, based on the similarity of its deduced proteins to those in *A. fumigatus* (see Additional file 2). Notably, this BGC contains a gene homologous to *sidF* from *A. fumigatus*, which encodes an acyl-CoA *N*-acyltransferase that catalyzes the biosynthesis of *cis*-AMHO (Additional file 1). Importantly, the predicted SidF protein in *P. roqueforti* shares 63.5% identity with its orthologue protein in *A. fumigatus* (Additional file 1). Therefore, this SidF orthologue may independently produce AMHO in *P. roqueforti*, explaining its presence in the copB-12 transformant. At this point, it is worth noting that under the experimental conditions employed in this study, neither fusarinine C nor its derivatives (fusarinine A and fusarinine B) were detected in any strain, including the wild-type fungus. Thus, another interesting possibility is that AMHO is a degradation product of these fusarinines (Fig. 2). Support for this proposal comes from previous studies on *A. nidulans* and *Mycelia sterilia*, organisms that produce fusarinine C and triacetylfusarinine C. In these species, it has been shown that these compounds can undergo intracellular hydrolysis, with the resulting degradation products being secreted into the extracellular medium [60, 61]. Although the functional characterization of fusarinines and their associated BGCs in *P. roqueforti* is beyond the scope of this study, this topic offers exciting opportunities for future research.

## Conclusion

In this study, we identify and characterize the coprogen biosynthetic gene cluster (*cop* BGC), responsible for synthesizing coprogen, a siderophore relevant to the fungus *P. roqueforti*. The identification and functional analysis of the *cop* BGC performed in this study provides the molecular basis for understanding the production of this important siderophore in *P. roqueforti*. Given the limited knowledge of siderophore biosynthesis mechanisms in this fungus, these findings will be valuable for future research on how *P. roqueforti* adapts to iron-limited environments, particularly in the context of blue-veined cheese, an ecological niche characterized by iron scarcity where siderophores may play a critical role in the fungus's survival and growth.

## Supplementary Information

Additional file 1. Scheme of the hypothetical fusarinine C BGC identified in *P. roqueforti*. The nomenclature of genes was according to those used in *Aspergillus fumigatus*. The *sidF* gene encoding the acyl-CoA *N*-acyltransferase, likely responsible for catalyzing the biosynthesis of *cis*-AMHO, is highlighted in blue. Alignment of SidF proteins from *A. fumigatus* and *P. roqueforti*. Identical amino acids are in red. The overall identity between both sequences is 63.5%.

Additional file 2. Analysis of the deduced proteins encoded by the fusarinine-type BGC from *Penicillium roqueforti*.

## Data Availability

The datasets used and/or analysed during the current study are available from the corresponding author on reasonable request.
