# Supplementary figures and images for: Molecular basis for the biosynthesis of the siderophore coprogen in the cheese-ripening fungus Penicillium roqueforti
Source: Biol Res. 2025 Jul 21;58:51. doi: 10.1186/s40659-025-00633-2 (PMC12278577; doi:10.1186/s40659-025-00633-2)

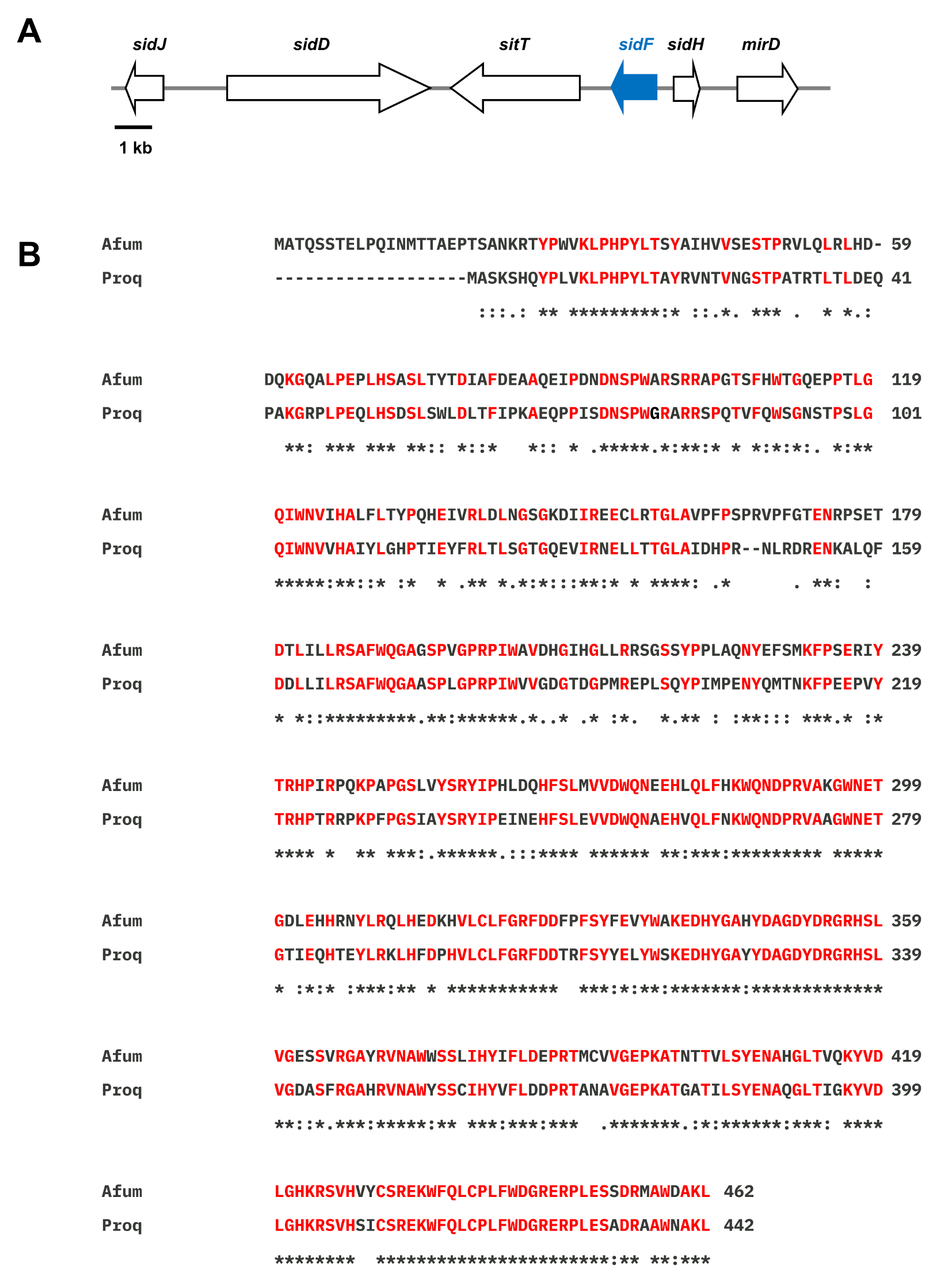

Supplement: Supplementary file 1 — Additional file 1. Scheme of the hypothetical fusarinine C BGC identified in P. roqueforti. The nomenclature of genes was according to those used in Aspergillus fumigatus. The sidF gene encoding the acyl-CoA N-acyltransferase, likely responsible for catalyzing the biosynthesis of cis-AMHO, is highlighted in blue. Alignment of SidF proteins from A. fumigatus and P. roqueforti. Identical amino acids are in red. The overall identity between both sequences is 63.5%. [file 40659_2025_633_MOESM1_ESM.png]
