# Supplementary material for: Molecular basis for the biosynthesis of the siderophore coprogen in the cheese-ripening fungus Penicillium roqueforti
Source: Biol Res. 2025 Jul 21;58:51. doi: 10.1186/s40659-025-00633-2 (PMC12278577; doi:10.1186/s40659-025-00633-2)
Supplement: Supplementary file 2 — Additional file 2. Analysis of the deduced proteins encoded by the fusarinine-type BGC from Penicillium roqueforti. [file 40659_2025_633_MOESM2_ESM.pdf]

Additional file 2: Analysis of the deduced proteins encoded by the fusarinine-type BGC from *Penicillium roqueforti*.

Name of the gene	Size of the deduced protein (amino acids)	Putative function	Closest homologue protein from a characterized BGC	Genbank accession number of homologue protein	Identity (%) with homologue protein
sidJ	350	Fusarinine C esterase	Fusarinine C esterase SidJ from *Aspergillus fumigatus*	Q4WF56	70
sidD	2,076	NRPS	SidD from *Aspergillus fumigatus*	Q4WF53	69
sitT	1,317	Transporter	ABC transporter SitT from *Aspergillus fumigatus*	EAL86625	77
sidF	442	Hydroxyornithine transacylase	Hydroxyornithine transacylase SidF from *Aspergillus fumigatus*	Q4WF55	64
sidH	272	Enoyl-CoA hydratase	Enoyl-CoA hydratase SidH from *Aspergillus fumigatus*	Q4WF54	55
mirD	593	Transporter	Fusarinine C transporter MirD from *Aspergillus fumigatus*	Q4WF51	54
